# Neutrophil-lymphocyte ratio predict outcome of upper gastrointestinal bleeding in emergency

**DOI:** 10.3389/fmed.2024.1366715

**Published:** 2024-08-08

**Authors:** Xinyi Chen, Xinqun Li, Guangju Zhao, Wen Xu

**Affiliations:** ^1^Department of Emergency, The First Affiliated Hospital of Wenzhou Medical University, Wenzhou, China; ^2^School of Basic Medical Sciences, Wenzhou Medical University, Wenzhou, China

**Keywords:** upper gastrointestinal bleeding, neutrophil-lymphocyte ratio, outcome, Glasgow-Blatchford score, full Rockall score

## Abstract

**Background:**

The neutrophil-lymphocyte ratio (NLR) is a simple marker of systemic inflammatory responses. The present study aims to evaluate the prognostic significance of the NLR on admission day in predicting outcomes for patients with upper gastrointestinal bleeding (UGIB), which is a prevalent medical emergency.

**Methods:**

726 patients who were admitted to our clinic between January 2019 and December 2022 diagnosed with UGIB, and who underwent necessary examinations, were included in the study. The patients’ Glasgow-Blatchford Score (GBS), Full Rockall Score (FRS), and NLR levels were calculated at the first admission. Outcomes were defined as in-hospital mortality, need for blood transfusion, surgical treatment and endoscopic therapy. Patients were categorized into four groups using NLR quartile levels to compare their clinical characteristics, Glasgow Blatchford Score, Full Rockall Score levels, and prognosis. Secondary, we modified FRS and GBS by adding NLR, respectively. We used area under the receiver operating characteristic curve (AUROC) to assess the accuracy of risk prediction for NLR, NLR-GBS, and NLR-FRS improved models.

**Results:**

Of 726 patients, 6% died in hospital, 23.9% received endoscopic interventon, 4.8% received surgical treatment, and 46.4% received transfusion therapy. Multifactorial logistic regression showed that a high level of NLR was a risk factor for death in patients with UGIB (*p* = 0.028). NLR, GBS, FRS, NLR-GBS, and NLR-FRS have sufficient accuracy in predicting inpatient mortality, endoscopic treatment, and transfusion treatment, and the differences are statistically significant (*p* < 0.05). In the comprehensive prediction of adverse outcomes, NLR-GBS has the highest AUROC, and in predicting inpatient mortality, NLR-FRS has the highest AUROC.

**Conclusion:**

For UGIB patients, a high NLR was strongly associated with high risk UGIB. Combined testing with the GBS and FRS can achieve good predictive results, which is valuable in guiding the pre-screening and triage of emergency nursing care and clinical treatment to ensure that patients receive rapid and effective treatment and improve the quality of care.

## 1 Introduction

Upper gastrointestinal bleeding (UGIB) is a commonly encountered emergency in clinical practice, with a mortality rate ranging from 2 to 15% (1, 2). Despite the advances in the management and endoscopic therapy strategies, the rate of mortality has not significantly improved (3, 4). Therefore, it is imperative to evaluate risk factors so that patients can be accurately stratified and disease activity and mortality can be assessed (5). Multiple systems have been designed and recommended by international guidelines. The most well-known systems are FRS, GBS, and AIMS65 (6, 7). However, certain limitations, such as complex parameters and the need for endoscopic findings, existed against the prevailing management of these scoring systems. Therefore, discovering simple, readily available markers for evaluating the prognosis of UGIB will be a challenging topic in UGIB practice.

When patients bleed, the body’s inflammatory factors are altered, and an inflammatory response occurs, affecting immune function (8). Peripheral blood inflammation index can be used to assist in the diagnosis of UGIB and reflect Outcomes (9). Most studies have shown that hematological indicators such as hemoglobin and RDW are associated with the prognosis of patients with upper gastrointestinal bleeding (10). The neutrophil-lymphocyte ratio (NLR) is a rapid and simple parameter of systemic inflammation and stress, which expresses the severity of the disease in the patients. In recent years, NLR has become new markers for predicting systemic inflammatory status due to their potential to evaluate the prognosis in critically ill patients (11), sepsis (12), hepatocellular carcinoma (13), gastric cancer (14), gastrointestinal bleeding in Henoch-Schonlein purpura (15), and so on. Furthermore, few studies have analyzed the usefulness of NLR in predicting the prognosis in UGIB patients.

In this study, we analyzed the expression level of NLR in patients with UGIB to clarify the value of NLR in prognosis and provide a new serological indicator for early risk detection. Exploring whether adding NLR to GBS and FRS can improve predictive efficacy. To investigate whether the addition of NLR to GBS and FRS can improve the predictive efficacy, aiming to help healthcare workers to be able to make a quick and simple supplementary comprehensive assessment of the severity and prognosis prediction of patients.

## 2 Materials and methods

### 2.1 Population and study design

This single-center retrospective observational study was administered in upper gastrointestinal bleeding patients who were admitted to the emergency department of the First Affiliated Hospital of Wenzhou Medical University between January 2019 and December 2022; 726 adult patients (aged ≥18 years) who conformed to the diagnostic standard of the expert consensus on emergency diagnosis and treatment of acute upper gastrointestinal bleeding were enrolled in this study. We excluded patients with a reference standard that included (1) Bleeding caused by surgical factors such as trauma, (2) missing baseline data. The enrolled participant was undergone endoscopy. The research was conducted in accordance with the principle of the Declaration of Helsinki and approved by the Medical Ethics Committee established in the First Affiliated Hospital of Wenzhou Medical University, China (KY2021-R063).

### 2.2 Data collection

Based on the existing work on this subject, a data questionnaire was designed to retrospectively collect general clinical information and laboratory data of patients at the time of admission from the electronic medical record system, including age, gender, previous medical history, heart rate, systolic blood pressure, hemoglobin, initial symptoms, related medication history (non-steroidal anti-inflammatory drugs), blood transfusion needs, endoscopic treatment, and in-hospital death, etc. NLR is defined as neutrophil count (10^9^/L) /lymphocyte count (10^9^/L), which is collected when the patient is first admitted to the hospital and has not received any treatment. Outcomes were defined as in-hospital mortality, need for blood transfusion, surgical treatment and endoscopic therapy. Endoscopic therapy was defined as embolization, ligation, sclerotherapy and application of hemostatic clips within 24 h after admission. GBS, FRS, and NLR levels were calculated at first admission, and GBS and NLR were calculated by the investigator.

### 2.3 Statistical analysis

The quantitative variables that conformed to normal distribution were expressed as means ± standard deviation ( $\bar{X}\pm S$ ) and the skewed distribution data were expressed as [M*(P25, P75)], and comparisons were made using a t-test and rank sum test, respectively. Data analyses were performed using SPSS 22.0. The chi-square test was used to compare two groups, and the Kruskal-Wallis test was used to compare multiple groups. Univariate and multivariate logistic regression analyses were performed on the influencing factors. The receiver–operator curve (ROC) was plotted to assess the predictive value of variables for outcomes. The regression model was used to establish the NLR-GBS combined model and the NLR-FRS combined model, and the area under the ROC curve compared the prediction accuracy of each scoring system and NLR for in-hospital mortality, Endoscopic interventon, surgery, transfusion therapy and composite of adverse outcome, *p* < 0.05 was considered statistically significant.

## 3 Results

### 3.1 Baseline characteristics and clinical outcomes

A total of 726 eligible patients admitted to the emergency department during the study period were included and categorized into survivor (*n* = 682) and non-survivor groups (*n* = 44), based on their eventual outcome. The differences in demographic characteristics between these groups and the comparative results are presented in Table 1.

**Table 1:** Baseline characteristics of patients enrolled in the study.

Clinical characteristic	Non-survivor (*n* = 44)	Survivor (*n* = 682)	*P*-value
Male gender (*n*, %)	38 (86.4)	531 (77.9)	0.183
Age (year)	60.5 (49.25, 68.5)	60 (48, 70)	0.896
Haematemesis (*n*, %)	31 (70.5)	364 (53.4)	0.027
Hematochezia (n, %)	28 (63.6)	532 (78)	0.028
Syncope (*n*, %)	5 (11.4)	86 (12.6)	0.809
Cause of bleeding (*n*, %)			<0.001
Esophagogastric variceal bleeding (*n*, %)	26 (59.1)	194 (28.4)	
Peptic ulcer hemorrhage (*n*, %)	7 (15.9)	316 (46.3)	
Coagulation abnormalities (*n*,%)	1 (2.3)	10 (1.5)	
Mallory-Weiss syndrome (*n*,%)	2 (4.5)	28 (4.1)	
Neoplastic factor (*n*,%)	5 (11.4)	50 (7.3)	
Acute gastric mucosa bleeding (*n*, %)	3 (6.8)	50 (7.3)	
Anastomotic bleeding (*n*, %)	0 (0)	13 (1.9)	
Other factors (*n*, %)	0 (0)	21 (3.1)	
Medication history (*n*, %)	6 (13.6)	107 (15.7)	0.716
Complications (*n*, %)	43 (97.7)	558 (81.8)	0.007
Smoking history (*n*, %)	17 (38.6)	172 (25.2)	0.142
Drinking history (*n*, %)	17 (38.6)	198 (29)	0.176
Gastrointestinal ulcer history (*n*, %)	2 (4.5)	89 (13)	0.099
UGIB history (*n*, %)	13 (29.5)	166 (24.3)	0.437
Systolic blood pressure (mmHg)	112 (98.25, 125.75)	120 (106, 135)	0.009
Heart rate (bpm)	99.5 (85.25, 109)	88.5 (78, 102)	0.008
Hemoglobin (g/L)	77 (57, 96.75)	87 (65.8, 108.3)	0.025
Blood urea nitrogen (mmol/L)	7.85 (5.6, 13.35)	9.45 (6.3, 13.8)	0.355
NLR	5.6 (3.54, 11.73)	4.14 (2.57, 7.03)	0.002
Transfusion therapy (*n*, %)	32 (72.7)	305 (44.7)	<0.001
Endoscopic intervention (*n*, %)	10 (22.7)	164 (24)	0.842
Surgery (*n*, %)	1 (2.3)	34 (5)	0.416
FRS (score)	6 (4, 6)	3 (2, 5)	<0.001
GBS (score)	12 (10, 14)	10 (7, 12)	<0.001

The age of the patients in the survival and non-survivor groups was 60 (48, 70) and 60.5 (49.25, 68.5), respectively, and the difference was not statistically significant (*p* ≥ 0.05). Concerning the laboratory data, the levels of Systolic blood pressure [112 (98.25, 125.75) vs. 120 (106, 135); *p* = 0.009] and hemoglobin [77 (57, 96.75) vs. 87 (65.8, 108.3); *p* = 0.025] in the non-survivor group were significantly lower, whereas the levels of heart rate [99.5 (85.25, 109) vs. 88.5 (78, 102); *p* = 0.008] was markedly higher than those of the survivor group. For bioindicators, the levels of NLR [5.6 (3.54, 11.73) vs. 4.14 (2.57, 7.03); *p* = 0.002] in the non-survivor group were markedly elevated than those in the survivor group. Otherwise, there was no significant difference in blood urea nitrogen levels between the survivor and non-survivor groups. Patients in the non-survivor group presented poorer prognoses with the outcomes of full Rockall scores [6 (4, 6) vs. 3 (2, 5); *P*<0.001] and Glasgow Blatchford scores [12 (10, 14) vs. 10 (7, 12); *P* <0.001]. Nevertheless, we failed to observe significant differences in terms of gender, smoking, and alcohol history.

### 3.2 Analysis of UGIB patients grouped by NLR quartiles

According to the NLR quartile level, they were divided into G1 group (NLR <2.618, 181 cases), G2 group (2.618 ≤ NLR <4.27, 181 cases), G3 group (4.27 ≤ NLR <7.196, 183 cases), and G4 group (NLR ≥7.196, 181 cases), and the differences of death, age, heart rate, and urinary urea were statistically significant among the groups. All patients were categorized into the high NLR group (*n* = 363) and low NLR group (*n* = 363) based on the median NLR of 4.27 as the cut-off value. Relatively more patients died, received endoscopic treatment, and were treated with blood transfusion within the high NLR group compared to the low NLR group, and the difference was statistically significant (*p* < 0.01). As the NLR values increased, the values of GBS and FRS also gradually increased, and the difference was statistically significant (*p* < 0.05) (Table 2).

**Table 2:** Comparison of clinical data of patients with different levels of NLR.

Clinical characteristic	1st quartile (*n* = 181)	2nd quartile (*n* = 181)	3rd quartile (*n* = 183)	4th quartile (*n* = 181)	*P*-value
NLR range	NLR<2.618	2.618 ≤ NLR<4.27	4.27 ≤ NLR<7.196	NLR ≥ 7.196	
Age (year)	56 (42,66.5)	60 (51,69)	62 (49.75,71.25)	62 (50,73.5)	0.001
Male gender (*n*, %)	138 (76.2)	140 (76.9)	146 (80.2)	145 (80.1)	0.706
Mortality (*n*, %)	4 (2.2)	8 (4.4)	16 (8.8)	16 (8.8)	0.015
Haematemesis (*n*, %)	67 (37)	92 (50.5)	111 (61)	125 (69.1)	<0.001
Hematochezia (*n*, %)	156 (86.2)	146 (80.2)	134 (73.6)	124 (68.5)	<0.001
Syncope (*n*, %)	15 (8.3)	22 (12.1)	30 (16.5)	24 (13.3)	<0.128
Cause of bleeding (*n*, %)					0.026
Esophagogastric variceal bleeding (*n*, %)	34 (18.8)	59 (32.6)	59 (32.2)	68 (37.6)	
peptic ulcer hemorrhage (*n*, %)	98 (54.1)	72 (39.8)	85 (46.4)	68 (37.6)	
Coagulation abnormalities (*n*, %)	4 (2.2)	3 (1.7)	3 (1.6)	1 (0.6)	
Mallory-Weiss syndrome (*n*, %)	7 (3.9)	12 (6.6)	3 (1.6)	8 (4.4)	
Neoplastic factor (*n*, %)	12 (6.6)	12 (6.6)	13 (7.1)	18 (9.9)	
Acute gastric mucosa bleeding (*n*, %)	17 (9.4)	13 (7.2)	13 (7.1)	10 (5.5)	
Anastomotic bleeding (*n*, %)	4 (2.2)	3 (1.7)	3 (1.6)	3 (1.7)	
Other factors (*n*, %)	5 (2.8)	7 (3.9)	4 (2.2)	5 (2.8)	
Medication history (*n*, %)	17 (9.4)	25 (13.7)	34 (18.7)	37 (20.4)	0.016
Complications (*n*, %)	146 (80.7)	153 (84.1)	151 (82.5)	152 (84)	0.807
Smoking history (*n*, %)	40 (22.1)	56 (30.8)	47 (25.8)	46 (25.4)	0.309
Drinking history (*n*, %)	48 (26.5)	56 (30.8)	60 (33)	60 (33)	0.551
Gastrointestinal ulcer history (*n*, %)	23 (12.7)	26 (14.3)	24 (13.2)	18 (9.9)	0.639
UGIB history (*n*, %)	45 (24.9)	37 (20.0.3)	51 (28)	46 (25.4)	0.392
Systolic blood pressure (mmHg)	121 (107.5,36.5)	121 (106,136.25)	119 (105,132.25)	116 (103.5,134)	0.35
Heart rate (bmp)	86 (79,100.5)	87 (76,100)	90 (80.75,103)	91 (80,104)	0.019
Hemoglobin (g/L)	92 (68.5,115.5)	86.5 (64.75,106)	86 (63, 108)	85 (64,103.5)	0.152
Blood urea nitrogen (mmol/L)	8.2 (5.1,11.6)	9.1 (5.98,3.3)	9.75 (6.6,14.83)	10.7 (7,15.85)	<0.001
Transfusion therapy (*n*, %)	63 (34.8)	81 (44.5)	88 (48.4)	105 (58)	<0.001
Endoscopic intervention (*n*, %)	27 (14.9)	46 (25.4)	45 (24.6)	56 (30.9)	0.004
Surgery (*n*, %)	10 (5.5)	8 (4.4)	8 (4.4)	9 (5.0)	0.951

### 3.3 Boxplot analysis of NLR and severity scores

**Figure 1:**
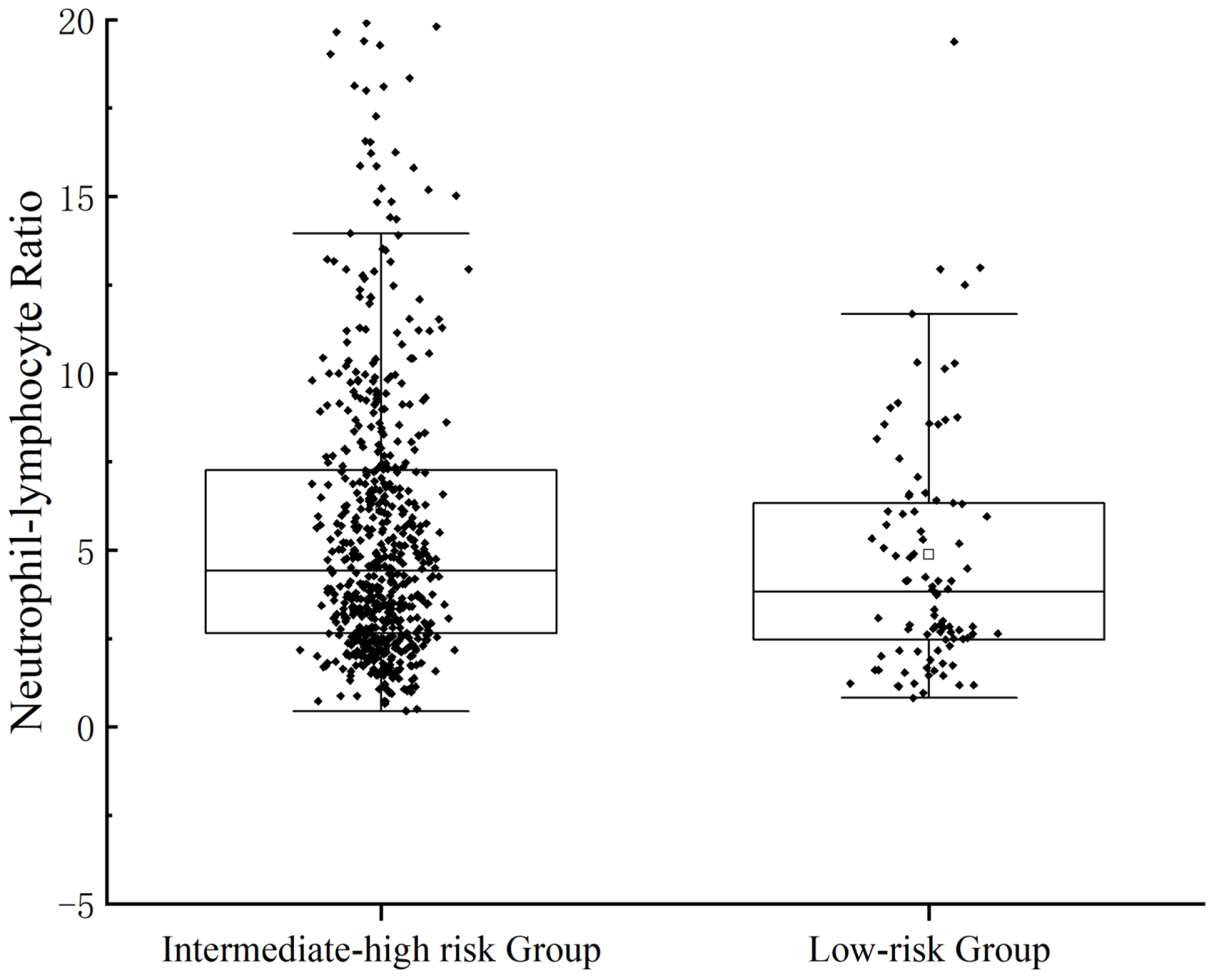
Patients’ NLR levels were categorized for severity. The correlation between NLR and the severity of UGIB was assessed by GBS.

**Figure 2:**
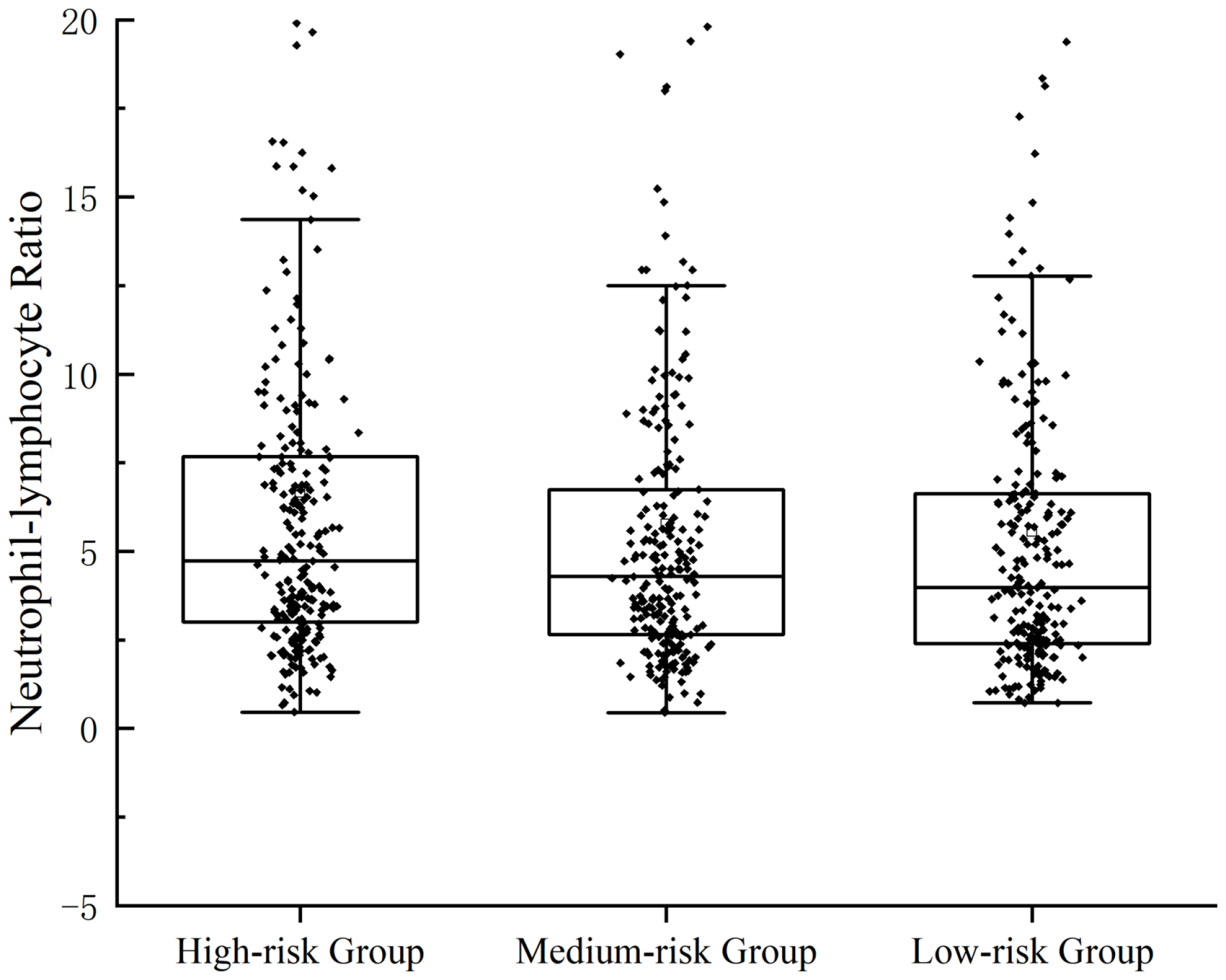
Patients’ NLR levels were categorized for severity. Correlation between NLR and the severity of UGIB, which was assessed by FRS.

Scatterplots were distributed to describe the correlation between NLR and the severity of UGIB. Rockall score (16) and Glasgow-Blatchford score (17) were used to score the enrolled patients, respectively; Rockall score 0–2 was categorized as a low-risk group, 3–4 was categorized as an intermediate-risk group, and ≥ 5 was categorized as a high-risk group; GBS <6 was categorized as a low-risk group, and ≥ 6 was a categorized as an intermediate-high-risk group. In the GBS subgroup, the NLR of patients in the medium-high-risk group was significantly higher than that of the low-risk group (*p* = 0.043); in the FRS subgroup, there was statistical difference in NLR among the three groups (*p* = 0.043) (Figures 1, 2).

### 3.4 Risk factors related to mortality

The univariate logistic regression analysis revealed that comorbidity, vomiting blood, blood in stool, heart rate, systolic blood pressure, hemoglobin levels, and NLR ≥ 4.27 were significantly associated with the occurrence of death in patients with upper gastrointestinal bleeding. These variables were subsequently included in a multifactorial logistic regression model to further analyze their impact on adverse outcomes in these patients (Tables 3, 4).

**Table 3:** Univariate analysis of risk factors affecting mortality from upper gastrointestinal bleeding.

Variable	β	*P*-value	OR	95% C.I.
Male gender	0.588	0.19	0.555	0.23–1.338
Age (year)	0.005	0.628	1.005	0.986–1.024
Systolic blood pressure (mmHg)	−0.023	0.003	0.987	0.963–0.992
Heart rate (bpm)	0.019	0.009	1.019	1.005–1.034
Hemoglobin (g/L)	−0.013	0.025	0.987	0.976–0.998
Haematemesis (*n*, %)	0.734	0.03	0.48	0.247–0.933
Hematochezia (*n*, %)	0.706	0.031	2.027	1.068–3.845
Syncope (*n*, %)	0.118	0.809	1.126	0.432–2.934
Medication history (*n*, %)	0.164	0.716	1.179	0.486–2.857
Complications (*n*, %)	2.257	0.026	0.105	0.014–0.767
Smoking history (*n*, %)	0.622	0.053	0.537	0.286–1.009
Drinking history (*n*, %)	0.431	0.179	0.65	0.346–1.219
BUN (mmol/L)	0.001	0.967	1.001	0.965–1.038
NLR ≥ 4.27	1.039	0.003	2.828	1.432–5.583

**Table 4:** Logistic regression model and the odds ratio of predictors.

Variable	β	*P*-value	OR	95% C.I.
Systolic blood pressure (mmHg)	−0.012	0.06	0.998	0.976–1.001
Heart rate (bpm)	0.017	0.022	1.017	1.002–1.032
Hemoglobin (g/L)	−0.012	0.06	0.998	0.976–1.001
Haematemesis (*n*, %)	−0.589	0.12	0.555	0.564–1.166
Hematochezia (*n*, %)	−0.589	0.12	0.555	0.564–1.166
Complications (*n*, %)	2.203	0.031	9.052	1.225–66.897
NLR ≥ 4.27	0.793	0.028	2.209	1.089–4.482

### 3.5 Pearson correlation analysis of NLR compared with GBS and FRS

The Pearson correlation analysis was conducted to examine the relationship between NLR and both GBS and FRS. The results revealed a positive correlation between NLR and GBS (*r* = 0.112, *p* < 0.05), as well as a positive correlation between the NLR and FRS (*r* = 0.094, *p* < 0.05) (Table 5).

**Table 5:** Pearson correlation analysis of NLR compared with GBS and FRS.

	NLR	
	r	*P*-value
GBS	0.112	0.003
FRS	0.094	0.012

### 3.6 Predictive performance of different scoring systems for mortality in patients with UGIB

**Figure 3:**
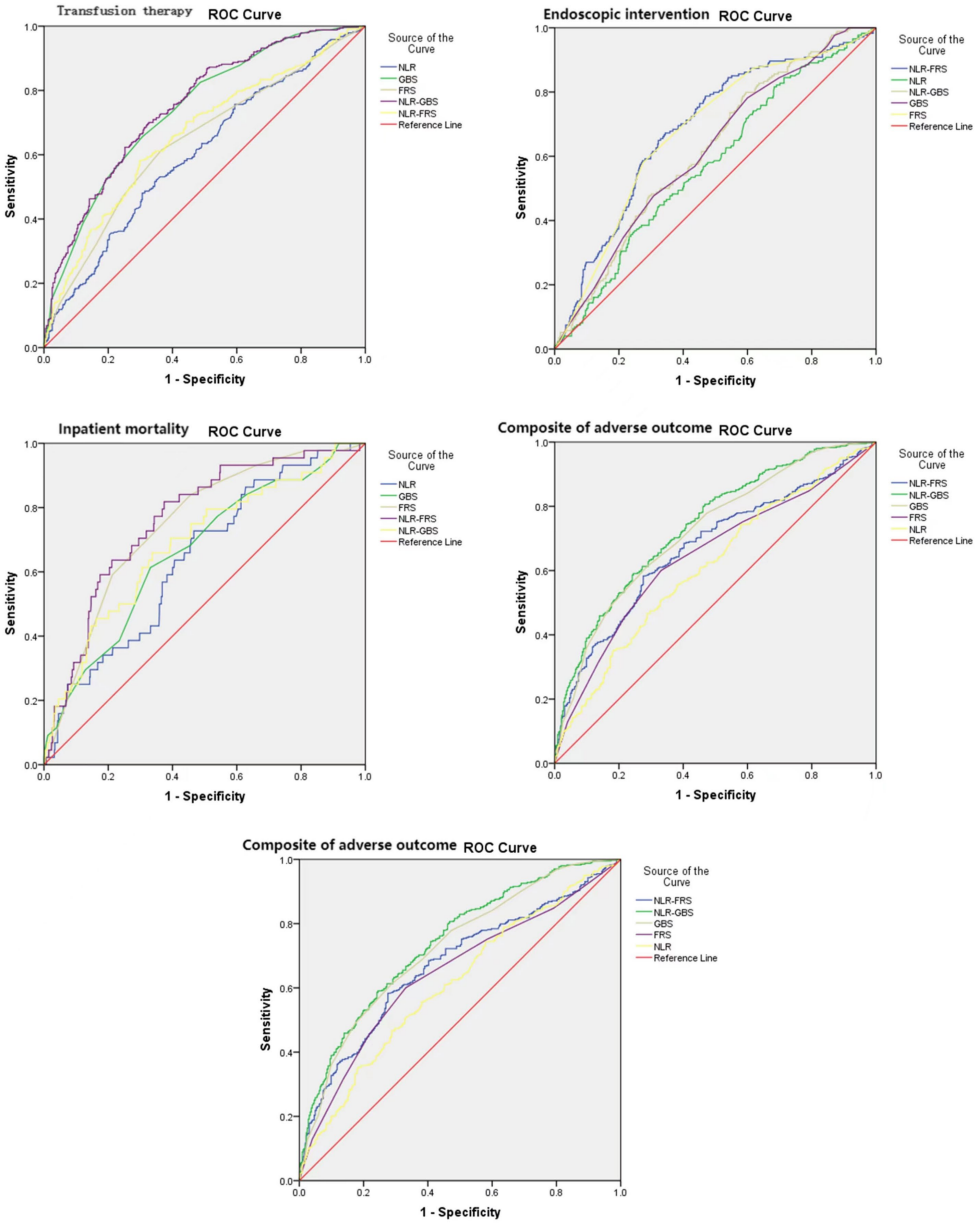
Reciever-operating characteristic curves (AUROCs) for the NLR, GBS, FRS, NLR-GBS, NLR, in predicting in-hospital mortality, transfusion therapy, endoscopic therapy, and composite of adverse outcome.

The ROC curve analysis was performed using the occurrence of in-hospital mortality in UGIB patients as the status variable (non-survival = 1, survival = 0), and NLR, GBS, FRS, GBS + NLR as the test variables. The results revealed that there was a statistically significant difference in AUC between FRS + NLR and FRS (*Z* = 2.033, *p* = 0.042). Additionally, the AUC for GBS + NLR was found to be higher than that for GBS with a statistically significant difference (*Z* = 20.072, *p* = 0.0383) (Table 6; Figure 3).

**Table 6:** ROC curve parameters.

	AUC	*P*-value	95%C.I.	Sensitivity	Specificity	Yoden index
**Inpatient mortality**						
NLR	0.640	0.002	0.562–0.719	0.727	0.534	0.261
GBS	0.662	<0.001	0.580–0.744	0.614	0.717	0.283
FRS	0.747	<0.001	0.678–0.815	0.841	0.543	0.384
NLR-GBS	0.687	<0.001	0.603–0.770	0.295	0.793	0.088
NLR-FRS	0.763	<0.001	0.695–0.832	0.818	0.626	0.444
**Transfusion therapy**						
NLR	0.603	<0.001	0.562–0.644	0.481	0.694	0.175
GBS	0.741	<0.001	0.705–0.776	0.825	0.514	0.339
FRS	0.639	<0.001	0.598–0.679	0.614	0.635	0.249
NLR-GBS	0.750	<0.001	0.716–0.785	0.700	0.674	0.374
NLR-FRS	0.660	<0.001	0.621–0.7	0.582	0.702	0.284
**Endoscopic intervention**						
NLR	0.575	0.003	0.527–0.622	0.816	0.317	0.133
GBS	0.616	<0.001	0.570–0.663	0.782	0.400	0.182
FRS	0.678	<0.001	0.633–0.723	0.580	0.725	0.305
NLR-GBS	0.619	<0.001	0.574–0.665	0.799	0.406	0.205
NLR-FRS	0.687	<0.001	0.642–0.731	0.600	0.527	0.127
**Composite of adverse outcome**						
NLR	0.611	<0.001	0.58–0.652	0.467	0.179	0.712
GBS	0.723	<0.001	0.686–0.760	0.613	0.712	0.325
FRS	0.645	<0.001	0.605–0.685	0.600	0.669	0.269
NLR-GBS	0.739	<0.001	0.703–0.775	0.591	0.755	0.346
NLR-FRS	0.670	<0.001	0.631–0.709	0.583	0.724	0.307
**Surgery**						
NLR	0.482	0.734	0.386–0.580	0.343	0.719	0.062
GBS	0.554	0.278	0.464–0.645	0.600	0.538	0.138
FRS	0.453	0.350	0.342–0.565	0.171	0.915	0.086
NLR-GBS	0.562	0.212	0.473–0.652	0.800	0.372	0.172
NLR-FRS	0.541	0.411	0.438–0.644	0.800	0.372	0.172

## 4 Discussion

Upper gastrointestinal bleeding is a common disease in the emergency department, with various etiologies and presentations. It is defined as blood loss originating proximal to the ligament of Treitz, in the esophagus, stomach, or duodenum (18). UGIB exhibits a substantial morbidity and mortality rate; therefore, early assessment and prediction of UGIB condition hold paramount clinical significance for enhancing patient prognosis. Currently, various scoring systems are employed to evaluate the condition and prognosis of UGIB patients (19). However, these systems possess inherent subjectivity, necessitating the urgent development of safer, more efficient, and objective biochemical markers that can aid in early prognostic prediction for UGIB patients. Such markers would greatly assist in guiding timely treatment interventions for UGIB.

The cost-effectiveness and widespread use of NLR as a composite inflammatory marker, reflecting the interplay between neutrophil and lymphocyte immune responses, make it a valuable tool for disease diagnosis. Elevated levels of NLR may indicate an increase in various pro-inflammatory cytokines concentration (20). More research evidence confirms the value of NLR in the assessment of disease and prognosis of cirrhosis (21), hepatocellular carcinoma (13), gastric cancer (14), allergic purpura combined with gastrointestinal hemorrhage, and ischemic stroke (22), etc., and Makay et al. found that the NLR values of patients with UGIB were significantly higher than those of patients without gastrointestinal hemorrhage (15).

The expression of NLR was significantly elevated in the non-survivor group with UGIB (*p* < 0.001). Moreover, it was observed that an admission peripheral blood NLR ≥4.27 independently contributed to the risk of in-hospital mortality in individuals with UGIB (*p* = 0.028). The NLR value of patients with UGIB in this study was divided into quartiles, and the clinical characteristics and prognosis were compared among patients in G1-4 groups. The results demonstrated a progressive increase in mortality rates from the G1 to G4 group (G1-4 groups, 2.2% vs. 4.4% vs. 8.8% vs. 8.8%, *p* = 0.015). Moreover, there was a gradual rise in the proportion of blood transfusion treatment from the G1 to G4 group (G1-4 groups, 34.8% vs. 44.5% vs. 48.4% vs. 58%, *p* < 0.001), along with an increasing trend in the proportion of endoscopic therapy (G1-4 groups, 14.9% vs. 25.4% vs. 24.6% vs. 30.9%, *p* = 0.004). These findings are consistent with those reported by Ramazan et al. (9). UGIB are affected by various stress factors and excessive bleeding, changes in levels of inflammatory factors in blood vessels, aggravating local damage at the bleeding site. Inflammatory reactions run through the entire course of the disease, and sustained inflammatory reactions are important factors that make it difficult for patients to stop bleeding, and even lead to death. The persistent inflammatory response is a crucial factor contributing to difficulties in achieving hemostasis and even mortality (23, 24). Koseoglu (25) and Kong (26) et al. reported that leukocyte values were higher in the group that died of UGIB than in the group that survived. Scholar Dertli found that high NLR level on admission was an independent risk factor for length of hospitalization and death in patients with non-variceal upper gastrointestinal bleeding (9), and high leukocyte values could be a predictor of high morbidity and mortality in patients with UGIB and could reflect the severity of bleeding and blood loss in patients (27, 28).

The most prevalent cause of UGIB is peptic ulcer, followed by bleeding from esophageal and gastric varices. This finding aligns with previous reports (29, 30).The level of NLR in patients presenting with UGIB demonstrated a significant correlation with the etiology of the hemorrhage (*p* = 0.026). Furthermore, our study revealed a progressive increase in the proportion of patients experiencing esophagogastric variceal bleeding (EGVB) across G1-4 groups. EGVB can induce the production of various inflammatory mediators, thereby promoting the development of acute inflammation (31), which in turn may lead to an acute systemic inflammatory response and increase the risk of mortality (32). Lin et al. demonstrated that the NLR independently predicted 30-day mortality in patients with acute decompensated cirrhosis (33), while Rice Jonathan et al. revealed a significant association between elevated NLR and increased all-cause mortality at 1 year in cirrhotic patients (34).

The expression level of NLR was higher in UGIB patients with a history of nonsteroidal anti-inflammatory drugs, and the proportion of patients with such history gradually increased from G1 to G4 (9.4% vs.13.7% vs. 18.7% vs. 20.4%, *p* = 0.016). Patients with UGIB mostly take NSAIDs (35), NSAIDs inhibit cyclooxygenase activity and decrease prostaglandin synthesis in the body, leading to a reduction in prostaglandin levels that can compromise the mucosal barrier and cause ischemia. The ischemic effect of NSAIDs induces inflammation, leukocyte migration, and vascular damage (9, 23).

The present study revealed a positive correlation between NLR and both GBS and FRS in patients with UGIB. In terms of predicting in-hospital mortality, the NLR-FRS model demonstrated superior performance compared to FRS (0.763 vs. 0.747, *p* = 0.042), while the NLR-GBS model outperformed GBS (0.687 vs. 0.662, *p* = 0.0383). Notably, the NLR-FRS model exhibited the highest AUC for predicting in-hospital mortality specifically in patients with UGIB, thereby providing a comprehensive reflection of changes in their clinical status.

There are some limitations in this study, such as this is a retrospective study, some patients were excluded due to the lack of necessary data, there may be a selection bias, and this is a single-center study, the results may not apply to other medical centers, the above conclusions need to be verified by additional studies.

## 5 Conclusion

In conclusion, the level of NLR is closely related to the prognosis of UGIB patients, which can play an early warning role for the risk of death in UGIB patients, and the combination with GBS and FRS can significantly improve the prediction efficacy, which can help to guide the clinic and nursing care in the early stage of risk stratification and the development of therapeutic care plan for the patients. With the advantages of simplicity, speed, and low cost, NLR deserves to be popularized.

## Data availability statement

The raw data supporting the conclusions of this article will be made available by the authors, without undue reservation.

## Ethics statement

The studies involving humans were approved by the Clinical Research Ethics Committee of the First Affiliated Hospital of Wenzhou Medical University. The studies were conducted in accordance with the local legislation and institutional requirements. The participants provided their written informed consent to participate in this study. Written informed consent was obtained from the individual(s) for the publication of any potentially identifiable images or data included in this article.

## Author contributions

XC: Writing – original draft, Writing – review & editing, Data curation, Formal analysis. XL: Project administration, Resources, Supervision, Writing – original draft, Writing – review & editing. GZ: Conceptualization, Formal analysis, Supervision, Writing – review & editing. WX: Methodology, Project administration, Writing – review & editing.
